# Non-Fistulizing Perianal Disease in Crohn’s Disease: Clinical Significance, Pathogenesis, and Management Strategies

**DOI:** 10.3390/jcm14248811

**Published:** 2025-12-12

**Authors:** Inês Abreu Marques, Tiago Cúrdia Gonçalves, Cláudia Macedo, Pedro Campelo, José Cotter

**Affiliations:** 1 Gastroenterology Department, Unidade Local de Saúde do Alto Ave, 4835-044 Guimarães, Portugal; tiagogoncalves@ulsaave.min-saude.pt (T.C.G.); claudiamacedo@ulsaave.min-saude.pt (C.M.); pedrocampelo@ulsaave.min-saude.pt (P.C.); josecotter@ulsaave.min-saude.pt (J.C.); 2 Life and Health Sciences Research Institute (ICVS), School of Medicine, University of Minho, 4704-057 Braga, Portugal; 3 ICVS/3B’s—PT Government Associate Laboratory, 4710-057 Braga, Portugal

**Keywords:** Crohn’s disease, perianal Crohn’s disease, fissure, ulcer, skin tags, strictures, anal cancer, hemorrhoids

## Abstract

**Background:** Perianal involvement is a well-recognized manifestation of Crohn’s disease (CD). However, non-fistulizing perianal phenotypes remain underrecognized despite their significance in clinical practice and impact on patients’ quality of life. **Methods:** A narrative review of the literature up to September 2025 was conducted, with an emphasis on studies that differentiated between non-fistulizing and fistulizing lesions. **Results:** During the CD course, approximately 45% of patients with CD develop non-fistulizing perianal manifestations, including fissures, ulcers, strictures, and skin tags. These lesions may resolve spontaneously with the ongoing CD therapy or additional conservative measures, but some evolve into more complex conditions, with challenging management. Deep ulcers and strictures appear to be associated with a less favorable disease course. While biologic therapy has altered the overall course of CD, its role in treating non-fistulizing perianal Crohn’s disease (PCD) requires further understanding. Surgical intervention, which carries an increased risk of complications, is typically reserved for individuals who are refractory to other treatments. The potential association between non-fistulizing PCD and anal cancer remains uncertain. **Conclusions:** Non-fistulizing PCD is a clinically significant condition that requires early recognition and individualized management. Prospective studies with standardized lesion classification, careful monitoring of disease course, and evaluation of biologic therapies and biomarkers are needed to develop evidence-based strategies and improve patient outcomes on non-fistulizing PCD.

## 1. Introduction

Perianal disease is a well-recognized manifestation of Crohn’s disease (CD) with important implications for both diagnosis and management. While earlier reports indicated that perianal Crohn’s disease (PCD) could precede intestinal symptoms in as many as 30% of cases, more recent evidence on fistulizing PCD suggests this number may be substantially lower [1]. Even in a smaller percentage of cases, it can be the sole manifestation of the disease, highlighting its diagnostic relevance and the importance of its early recognition [2,3,4]. Clinical attention and research have been focused on fistulizing PCD due to its complexity and refractory nature. However, other non-fistulizing manifestations may be present [5].

Non-fistulizing PCD includes a heterogeneous spectrum of conditions that can range from mild to severe, including anal fissures, ulcers, strictures, or skin tags [6]. As a result, patients with on-fistulizing PCD may experience anal pain, discharge, dyschezia, or fecal incontinence [7]. These symptoms can result in considerable functional limitation in daily routine, adversely affecting psychological, social, and sexual well-being, and ultimately impairing patients’ quality of life [7,8]. Many of these conditions can be underrecognized in clinical practice, which delays their treatment. A study in a pediatric population showed that patients with fistulizing PCD received immunomodulatory or antibiotic therapies significantly earlier than those with non-fistulizing perianal phenotypes of CD [9].

Additionally, management of non-fistulizing PCD can be challenging due to the underlying inflammatory process of the disease and the lack of formal recommendations on management and treatment [10]. The available evidence is often derived from small, retrospective studies and subgroup analysis with few randomized and controlled prospective studies. Therefore, the objective of this review is to assess the existing evidence regarding non-fistulizing PCD, an area that remains underexplored and supported by limited literature. Furthermore, this work aims to identify gaps in current knowledge and outline directions for future investigation to improve clinical management.

## 2. Materials and Methods

A narrative review was conducted on the online database PubMed. Only articles published in English up to September 2025 were included. The search was performed using the following keywords: Crohn’s disease, perianal Crohn’s disease, fissures, ulcers, skin tags, strictures, anal cancer, and hemorrhoids. The inclusion criteria comprising publications addressing non-fistulizing PCD. Studies not directly relevant to the topic and those for which the full text was unavailable were excluded. Additional articles were identified through manual screening of reference lists from key reviews.

Clinical trials, observational studies, preclinical data, and key review articles were considered to ensure a broad and integrative understanding of the topic. Due to the scarcity and heterogeneity of the literature on this topic, as well as the scope of the narrative review format, no bias assessment or quantitative synthesis were performed.

Studies were reviewed to evaluate the epidemiology, natural history, clinical presentation, diagnosis, and management strategies, with particular attention to studies that differentiated between non-fistulizing and fistulizing perianal lesions. We acknowledge the inherent limitations of this format, including potential selection bias and subjectivity in the study inclusion process.

## 3. Epidemiology and Natural History

PCD is characterized by a heterogeneous group of lesions that affect the perianal skin and anal canal and can be classified as primary, secondary, or incidental lesions. Primary lesions are caused by a similar idiopathic inflammatory process as other gastrointestinal CD lesions [6,11]. Secondary lesions arise as complications from the primary lesions, particularly fistulas, abscesses, or strictures. Incidental lesions, by contrast, are not directly associated with CD, such as hemorrhoids [11].

The incidence, risk factors, and clinical course of non-fistulizing PCD are challenging to determine, as most studies focus on fistulizing PCD or often classify it as a single entity overlapping both phenotypes. In fact, patients with PCD can present with more than one perianal lesion, both fistulizing or non-fistulizing [12]. The association among several lesions and their misclassification or non-documentation in clinical practice makes the course of non-fistulizing PCD poorly known [13]. Peyrin-Biroulet et al. calculated a 30-year cumulative risk of 44.8% for developing non-fistulizing PCD [14]. Among the reported lesions, skin tags and fissures were the most frequently observed, with incidences of 4.6% to 19.1% and 27.3% to 30.1%, respectively, as described in Table 1 [12,14,15,16,17]. A limitation reported by Eglinton et al. was the assumption that all perianal disease was associated with CD, particularly fissures [15]. Furthermore, the variability in the reported incidence of lesions among the presented studies may be attributed to heterogeneity in study design, including retrospective and prospective studies, as well as population characteristics.

Non-fistulizing PCD has been associated with greater morbidity and is a predictor of a severe disease course overall [18]. Deep cavitating ulcers have been linked with poorer outcomes and greater negative impact on patients’ quality of life [19]. Keighley et al. demonstrated worse morbidity in patients with cavitating anorectal ulceration and rectal strictures, with 25% of patients presenting with non-healing lesions that led to proctectomy [2].

Established risk factors for PCD include colonic disease and young age at disease onset [20]. Anal ulcerations have been associated with the severity of luminal disease and are more frequent when distal colic and rectal involvement are present [12,21]. Although less frequent, PCD may also be present when luminal CD activity is limited to the small bowel [2,22,23]. Interestingly, the phenotype of luminal CD (inflammatory, structuring, or penetrating) does not correlate with the presence or type of perianal lesions [12].

## 4. Classification

Accurate classification of non-fistulizing PCD is essential for both research purposes and to guide management and follow-up strategies. Recognizing this need, Hughes et al. suggested a review of the Cardiff classification for PCD in 1992, comprising a standardized anatomical classification (Table 2) [24].

More recently, the American Gastroenterological Association (AGA) proposed a broader classification system, distinguishing between fistulizing disease (fistulas and abscesses) and non-fistulizing disease (anal fissures, anal ulcers, skin tags, anorectal strictures, and anal cancer) [6].

Horaist et al. assessed interobserver agreement on PCD diagnosis among members of the Société Nationale Française de Coloproctologie. Results showed moderate agreement on the description of skin tags, ulceration, and in the evaluation of inflammatory activity. However, other areas, such as description of the location of lesions on the anal canal and depth and extension of ulcerations, showed lower agreement. Additionally, the study did not distinguish fissures from ulcers [25]. Both are often described as a single entity, which may hinder the interpretation of the studies’ results.

For assessing disease activity, Irvine et al. proposed the clinical score Perianal Disease Activity Index (PDAI), which has since been adopted as a clinical and therapeutic endpoint in clinical research [26]. PDAI has undergone prospective validation in studies examining fistulizing phenotypes. However, its validity in evaluating inflammatory activity in non-fistulizing PCD remains to be defined [27,28,29].

Despite the availability of several classification systems, there appears to be no consensus regarding the optimal classification of non-fistulizing forms of PCD. The wide variety of features included across existing systems reflects uncertainty over which findings are specific to CD and which represent common proctologic conditions occurring in patients with CD [30].

## 5. Diagnosis

Clinicians should be aware of a patient’s history of perianal disease and inquire about current symptoms. A significant proportion of patients may be asymptomatic, and subtle changes can be the only indicator of disease. Patients may overlook or feel uncomfortable in describing these symptoms [8,27]. In the presence of perianal symptoms, for instance, anal/perianal pain, discharge, pruritus, incontinence, or rectal bleeding, a detailed history should take place, with bowel habit documentation as well as recent changes [31].

Physical examination is essential and often sufficient to establish a diagnosis of non-fistulizing PCD. However, distinguishing between skin tags and hemorrhoids from other lesions, such as abscesses, perianal fistulas, and anorectal strictures, may be a challenge [32]. Careful inspection of the perianal region, including palpation of the area and a digital rectal examination, can provide valuable information. In cases of severe pain and complex PCD, examination under anesthesia (EUA) may be required [10]. Furthermore, anoscopy is also an essential part of the examination, when feasible, and requires sedation in symptomatic patients to achieve adequate visualization of the anal canal [22]. When documenting lesions, it is essential to describe the number and location, and to actively look for inflammatory signs, purulent discharge, asymmetries, scarring, or bulging in the perianal area.

An association between ileocolonic disease activity and the development of perianal lesions has been observed, prompting endoscopic evaluation of inflammatory activity in patients with PCD who develop these lesions [12,21,33]. However, Buchmann et al. reported conflicting evidence, suggesting that the persistence and the healing of perianal lesions were unrelated to intestinal activity [34]. When planning endoscopic evaluation with bowel preparation to assess intestinal inflammatory activity, the potential risk of exacerbating symptoms of PCD should be carefully considered.

**Figure 1:**
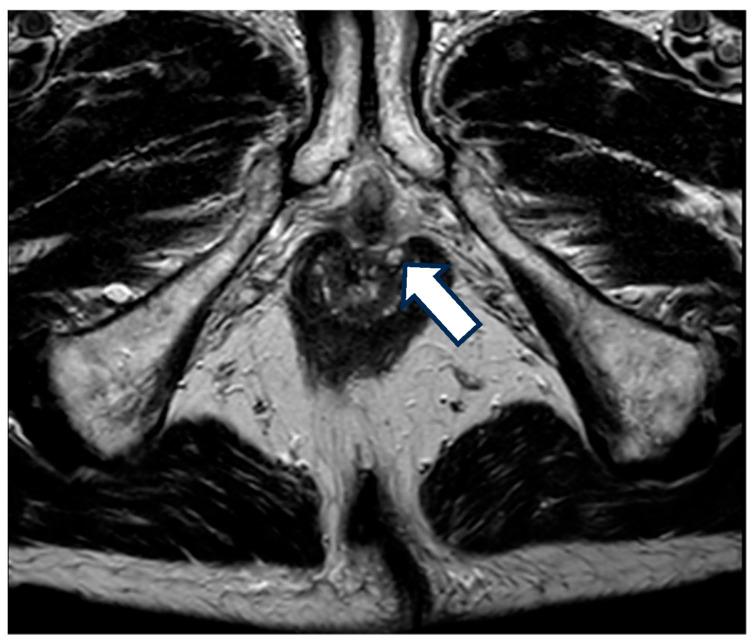
MRI with fistula. A 68-year-old patient with a 20-year history of Crohn’s disease and a prior diagnosis of perianal Crohn’s disease (PCD) presented with anal pain. Upon examination, a perianal ulceration approximately 3 cm in diameter was observed, and medical treatment with antibiotics was initiated. A pelvic MRI was requested for further characterization, which identified several fistulous tracts, without the identification of the location of the anal ulcer. The arrow in the image is pointing towards one of the fistulous tracts.

In addition to EUA, the European Crohn’s and Colitis Organization (ECCO), European Society of Gastrointestinal and Abdominal Radiology (ESGAR), the European Society of Pathology (ESP), and the International Bowel Ultrasonography Group (IBUS) 2025 consensus recommend cross-sectional imaging with pelvic magnetic resonance (MRI) or, if MRI not available, endoscopic ultrasound in the evaluation of PCD with good diagnostic accuracies and the ability to monitor therapeutic response for fistulas and abscess. Its role in non-fistulizing PCD, however, remains unclear [35,36]. A retrospective study performed by Garros et al. demonstrated that MRI failed to diagnose 95% of superficial ulcers, 87% of severe ulceration, and consistently failed to diagnose strictures, both reversible and severe irreversible anorectal strictures [37]. Therefore, imaging should be used in the clinical suspicion of fistulas and abscesses or to exclude this diagnosis in complicated non-fistulizing PCD, since these conditions are often associated [38], as illustrated in Figure 1.

In a retrospective study by Figg et al., perianal biopsies were performed in patients with and without perianal involvement from CD. None of the biopsies of patients without PCD had granulomas, whereas 77% with PCD had granulomas. Additionally, the patients with granulomas found on biopsies had more difficulty healing fissures and ulcers [22]. Taylor et al. investigated excisional biopsies of anal skin tags as an adjunct to rectal biopsy in patients with known CD. Granulomas were found in almost 30% of patients, and when present, were more numerous in skin tags than in biopsies of the rectal mucosa. No granulomas were found in skin tags from patients without CD [39]. However, there is insufficient evidence to support routine biopsy of perianal lesions for the histological confirmation of CD [19]. Nevertheless, this data may have clinical relevance in facilitating the early recognition of Crohn’s disease when luminal manifestations are absent.

In addition, it is essential to consider other conditions with a similar clinical presentation to PCD [13,19]. Differential diagnosis should include infectious diseases such as tuberculosis or sexually transmitted infections (human immunodeficiency virus—HIV, chlamydia, gonorrhea, syphilis, HPV infection), hidradenitis suppurativa, post-radiotherapy treatments, hematopoietic malignancy, among others [19]. Biopsy and bacteriological cultures can be valuable diagnostic tools for differential diagnosis [13,27]. In cases of atypical or non-healing skin tags, ulcerations, or long-standing and long-segment strictures, biopsy may be necessary to exclude malignancy [40,41]. Additionally, when perianal lesions are associated with a mass, it should raise suspicion of anal cancer [13].

## 6. Clinical Manifestations and Management

### 6.1. Fissures

Anal fissures typically result from hypertonicity of the anal sphincter and local trauma of the anoderm. They are mainly located in the posterior anal midline, with fewer cases in the anterior midline. Multiple, atypically located, painless or non-healing fissures should raise suspicion for the diagnosis of Crohn’s disease [11,42]. Although older literature described CD-related fissures as painless, recent studies report pain in 70% of individuals, often exacerbated by defecation [43]. Patients may also present other symptoms such as rectal bleeding, discharge, or pruritus [44,45].

Fissures may heal spontaneously with the underlying treatment of CD, with reported healing of 49% [21,46]. In a 10-year follow-up by Buchmann et al., 19% of patients had persistent fissures and 50% of patients developed induration and some degree of stenosis [34,43].

#### 6.1.1. Medical Treatment

Standard therapies used for non-CD fissures may be effective, with initial recommendations similar to those given to the general population [45]. Conservative measures include warm sitz baths, bowel regularization, and topical healing agents or analgesic ointments. In patients with fissures associated with diarrhea resulting from CD, the control of bowel movements is a particularly important factor to consider [10]. The role of agents that relax the anal sphincter is yet to be determined. Nitroglycerin, diltiazem, or botulinum toxin injection may be beneficial in patients with hypercontractile sphincters, but its effectiveness can be lower if diarrhea or proctitis are present [19].

#### 6.1.2. Surgical Treatment

Surgical treatment, such as fissurectomy or lateral internal sphincterotomy, is reserved for individuals with refractory fissures and is associated with poorer outcomes in the presence of active disease due to impaired healing [47,48]. Fleshner et al. performed surgical treatment in 8 of 46 patients presenting with fissures. Among these, three patients underwent lateral internal sphincterotomy, two underwent fissurectomy, and three received both procedures. The remaining 38 patients were managed with medical therapy. The authors reported one case of a post-procedure non-healing fissure following lateral internal sphincterotomy. Overall, surgery resulted in a higher initial healing rate compared with medical therapy. Despite treatment modality, six patients in each group, medical and surgical, ultimately required proctectomy due to persistent anal sepsis originating from the fissure site [43].

In a case series of 25 patients who underwent surgery for symptomatic fissures, 22 patients achieved healing within two months, whereas three patients experienced delayed or incomplete healing. During the follow-up, 11 patients developed anorectal complications, including fistulas, abscesses, strictures, recurrent fissure, and two cases required proctectomy [49].

Based on these findings, it appears that surgical treatment should be carefully considered, and less invasive measures should be prioritized.

### 6.2. Ulcers

**Figure 2:**
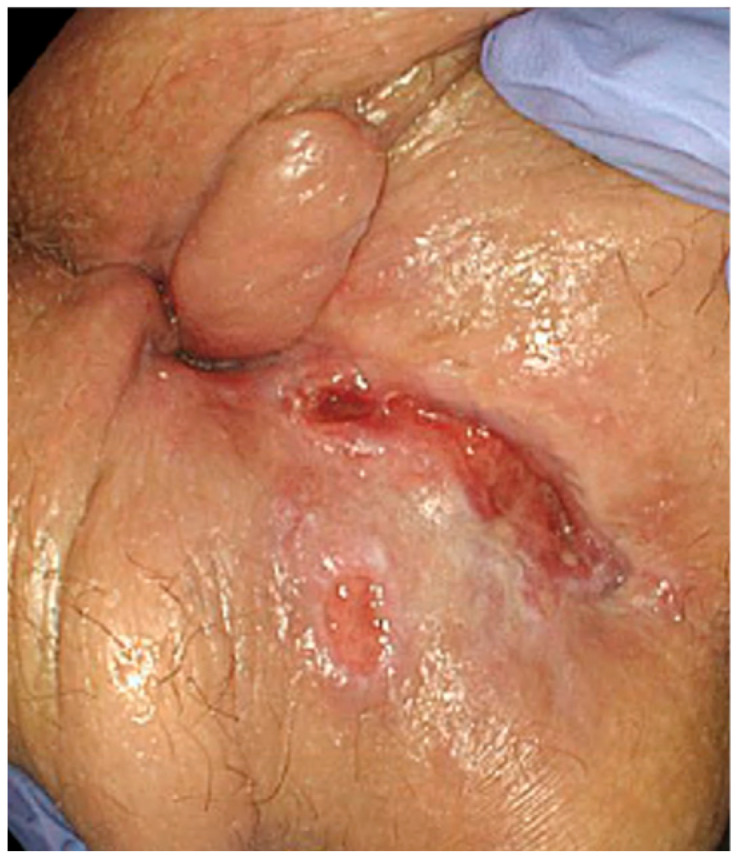
Patient presenting with anal ulceration in the posterior line with an associated skin tag.

Anal and perianal ulcers in CD are derived from deep inflammatory damage and extensive lymphoedema of the subcutaneous tissue. They can affect the anal canal, sphincter muscles, perianal tissues, and rectal wall, with increased risk of abscess, fistula, and stricture formation [11]. Symptoms are often more severe, with 56% of patients reporting unremitting anal pain [21]. An example of perianal ulcer is represented in Figure 2.

#### 6.2.1. Medical Treatment

Wallenhorst et al. reported that more than half of anal ulcerations were associated with fistulas, whereas 35.7% occurred in isolation. In addition, despite 43% of patients already being treated with biologic agents at referral, the authors reported that optimizing medical therapy by initiating, intensifying, or switching TNF-α antagonists significantly reduced the risk of an unfavorable course. The cumulative healing rates for anal ulcerations were 28%, 47%, 70% and 82% at 6 months and 1, 2, and 3 years, respectively [12].

The role of immunomodulators, such as azathioprine, remains to be determined. Cosnes et al. showed reduced active PCD and need for perianal surgery in patients with early introduction of azathioprine on disease onset, defined within 6 months of diagnosis [50]. Combination therapy of immunomodulators and TNF-α antagonists might be beneficial, but studies validating this benefit in non-fistulizing PCD are needed [51].

In a group of patients with PCD, including six with anal ulcers, treatment with ciprofloxacin (1000–1500 mg/day) plus metronidazole (500–1500 mg/day) led to symptomatic improvement within 12 weeks in most cases, with a high rate of resolution of pain. However, complete healing was achieved in only three patients, although the description of which lesions healed was not provided. Some patients had relapsing symptoms when discontinuing this therapy [52]. Besides this study, there is limited evidence on the use of antibiotics for non-fistulizing PCD. Still, antibiotics may provide symptomatic relief and be considered in addition to other therapies for temporary use.

#### 6.2.2. Other Treatment Options

A randomized controlled trial on a heterogeneous group of PCD patients showed that topical metronidazole 10% for a period of 4 weeks reduced anal pain and discharge compared with placebo, but without a significant reduction in PDAI [53]. In another study, topical metronidazole in the same dosage demonstrated potential benefits in symptom control and in reducing the PDAI score in patients with PCD, of whom five had fissures and one had both a fissure and a stricture [54].

In patients with a loss of response to other treatments, topical tacrolimus at an intermediate dosage showed improvement, with a reduction in ulceration depth and extension compared to placebo, although it did not result in complete healing in the four patients studied [55].

Hyperbaric oxygen therapy can be considered an alternative treatment for refractory lesions, as it is theorized that it may modulate the local perianal immune response. However, its role in PCD remains unclear [56,57]. Feitosa et al. reported promising results, with complete healing achieved in 65% of patients undergoing hyperbaric oxygen therapy for PCD that was refractory to medical treatment. However, that group of patients was heterogeneous, with both fistulizing and non-fistulizing PCD [58].

#### 6.2.3. Surgical Treatment

The surgical manipulation of non-healing ulcers is associated with worse outcomes and complications [59]. Lesions refractory to all medical measures may require fecal diversion, proctectomy, or proctocolectomy with permanent end ileostomy [19,40].

### 6.3. Skin Tags

AGA describes two types of skin tags in CD. One type is characterized by large, edematous, hard, and cyanotic skin tags, which are typically larger in size and usually arise from a healed anal fissure, ulcer, or hemorrhoids. And another type, often described as “elephant ear” tags, that are flat, soft, broad or narrow, and painless skin tags that can develop from perianal lymphedema [6,41]

A higher prevalence of the colonic CD phenotype (46.8%) has been described in this subset of patients [60]. Additionally, not all skin tags are associated with CD, as they can also occur in ulcerative colitis [41,60].

#### Surgical Treatment

Skin tags are usually persistent, with 68% of patients maintaining lesions during a 10-year follow-up performed by Buchamann et al. [34]. While patients should be reassured of its benign course, there is one documented case of malignant transformation of a skin tag associated with CD [61].

Routine removal is not recommended, since it is associated with impaired healing and possibly fecal incontinence due to the underlying active inflammation. Nevertheless, removal can be considered in “elephant ear” tags or fibroepithelial polypoid tags in the absence of proctitis, particularly if patients experience concerns related to image or hygiene [6].

### 6.4. Strictures

Strictures can develop in the rectum or anal canal because of ongoing rectal inflammation of CD or as a complication of previous lesions. Furthermore, they may be short, annular diaphragm-like strictures (<2 cm in length) or longer tubular strictures [6].

According to the Cardiff classification, S1 type strictures are reversible and relax under anesthesia. These conditions typically have a slight to moderate clinical impact and include examples such as anal canal spasm (S1a), low rectum membranous strictures (S1b), often resulting from circumferential superficial ulceration, and cases of spasm associated with severe pain but without identifiable sepsis (S1c). On the other hand, S2 type strictures are irreversible, failing to soften in response to anesthesia due to fibrosis arising from chronic inflammatory activity, previous lesions, or even conditions unrelated to Crohn’s disease, such as prior surgeries. S2 strictures include anal stenosis (S2a) and extra-rectal fibrotic strictures (S2b), the latter often secondary to perirectal extension of deep abscesses [24,62].

#### 6.4.1. Medical Treatment

Asymptomatic strictures generally require no intervention. If proctitis or anal canal inflammation is present, rectally administered topical agents represent the first-line therapeutic option, including 5-aminosalicylates and corticosteroids [62].

S2 or irreversible strictures are usually more symptomatic, with bloody stools, constipation, soiling, or fecal incontinence [63]. Symptomatic strictures may improve with medical therapy when active inflammation is present [63,64]. In a retrospective cohort of 102 patients with anorectal strictures, 59% of patients achieved stricture healing under biologic therapy. After stricture diagnosis, optimization of TNF-α antagonists, mainly infliximab, significantly reduced the risk of unfavorable progression. Still, about one third of patients experienced an unfavorable outcome, defined by persistent stricture or the need for permanent stoma or proctectomy. Disease duration <10 years, and presence of an anal fistula at the time of stricture diagnosis were associated with higher healing rates [64].

#### 6.4.2. Surgical or Endoscopic Treatment

In the absence of active inflammation, dilation can be considered for the treatment of strictures, taking into account the risk of incontinence and sphincter damage [40,42].

In a case series published by Alexander-Williams et al., five patients underwent dilation of 7 rectal strictures with coaxial balloons, metal dilators, or both techniques. Three patients remained asymptomatic after dilation at 7 months of follow-up. Two patients developed recurrent symptomatic stricture, requiring proctectomy. One patient developed septicemia within hours of dilation in the presence of active Crohn’s colitis, which was promptly resolved with antibiotic therapy [65].

Michelassi et al. observed 41 patients with isolated strictures. Eight were asymptomatic and did not receive any therapy, 39 procedures were performed in the 33 symptomatic patients—16 underwent mechanical dilations, while 17 underwent proctectomy or diverting stoma. No information regarding ongoing medical therapy was reported [66].

The Global Interventional Endoscopy IBD study group consensus recommends endoscopic treatment for anorectal strictures, including endoscopic balloon dilation, needle-knife endoscopic stricturotomy, or a combination of both methods, thereby avoiding the need for permanent diversion [67]. However, no clinical trials or prospective outcome data were found regarding the efficacy of dilation.

### 6.5. Biologic Therapy

Evidence supports the use of anti-TNF-a therapies on fistulizing PCD [5]. In PCD, a complex network of cytokines plays a key role in its pathophysiology, with IL-1β, IL-12, IL-6, and TNF-α being central mediators of inflammation. Ruffolo et al. demonstrated significantly higher levels of TNF-α and IL-6 in the presence of fistulas, with a correlation to PDAI. In contrast, strictures were associated with higher levels of IL-12 and significantly lower levels of IL-6. This may indicate the role of IL-12 in post-inflammatory repair mechanisms, and that IL-6 downregulation may occur once fibrosis becomes dominant [68]. In another study, Ruffolo et al. also demonstrated that elevated mucosal IL-6 and IL-12 levels predict the recurrence and the need for surgery in PCD. These findings suggest that targeting IL-6 and IL-12, in addition to TNF-α, may enhance treatment strategies [69]. They further emphasize the need for individualized treatment approaches tailored to specific disease contexts.

Wallenhorst et al. conducted a retrospective study on PCD patients, including 154 patients with anal ulcers and 49 with strictures. The results demonstrated that anti-TNF-α therapy (infiximab used more frequently than adalimumab) had significantly improved healing of anal ulceration, with a healing rate of 63% over a 65-week period. No significant differences were observed in the resolution of strictures. A better potential for resolution was observed in patients with high or complex fistulas in this study [46].

After infliximab induction, Bouguen et al. reported a complete response in 42.5% of patients with ulcers and 18.2% with strictures. After a median follow-up of 175 weeks, complete response rates increased to 72.3% and 54.5%, respectively. Long-term response for cavitating ulcers was positively associated with concomitant immunosuppressant use and older age. As half of the patients with complete stricture regression also underwent anal dilation, the real effect of infliximab in this setting is difficult to ascertain. Three patients with ulcers developed anal abscesses. Authors’ findings indicate that infliximab is generally well tolerated and effective in inducing and maintaining complete clinical responses in perianal ulcers [45].

Further randomized studies are needed to evaluate the efficacy and clinical response of anti-TNF-α and other biologic treatments in patients with non-fistulizing PCD.

### 6.6. Anal Cancer

Although a rare complication of PCD, there are case reports associating it with anal and rectal cancers, including adenocarcinoma or squamous cell carcinomas [70]. A recent systematic review by Wong et al. showed that patients with chronic complicated perianal fistulizing are at increased risk of cancers in the perianal region, near or involving perianal fistula tracts [20,71]. It is not clear whether patients with chronic non-fistulizing perianal phenotypes also have an increased risk of anal and rectal cancers [72]. Two cases of anal adenocarcinoma during stricture follow-up were observed by Brochard et al. [64]. On the other hand, strictures can delay or obscure diagnosis, as well as other confounding perianal lesions. A low threshold for biopsies should be maintained in the presence of new induration, persistent pain, or a change in drainage in chronic perianal disease. Due to the severity of this condition, as it can be asymptomatic or present with non-specific symptoms, some authors suggest that an anal cancer screening program could be justified in CD patients with anal and/or perianal involvement, like other high-risk groups for malignancy, such as HIV patients [73]. In that regard, in addition to the ECCO recommendations for HPV vaccination for young adults, some authors suggest a surveillance program for all patients with anal and/or perianal CD with proctological evaluation, including anoscopy and anal cytology with HPV testing, with optimal timings of surveillance to be determined in future studies [73,74]. Regarding treatment, AGA recommends that standard oncologic surgical principles and procedures should be followed as in non-CD patients [6].

### 6.7. Hemorrhoids

Hemorrhoids are not a manifestation of an underlying active inflammatory process of CD, but a common condition in the general population and in patients with CD. The first-line treatment for this condition consists of conservative measures, which are effective in most cases [75]. Conservative measures include a high-fiber diet, fiber supplements, and adequate fluid intake to promote soft, regular stools, along with warm sitz baths. In patients with diarrhea, symptom control is essential. Additionally, flavonoid therapy can be used as supportive treatment [76,77].

Surgical management has been associated with poor wound-healing, infection, and anal canal stenosis [48]. However, evidence remains conflicting. Wolkomir and Luchtefeld reported few complications in selected patients with quiescent CD and symptomatic hemorrhoids unresponsive to conservative measures. While one patient required proctectomy 15 years after hemorrhoidectomy, the authors concluded, based on their findings, that hemorrhoidectomy could be considered in highly selected cases [49].

In a retrospective study by D’Ugo et al., 45 patients with CD who underwent hemorrhoidectomy were reviewed. Some patients had a previous CD diagnosis, while in others, the diagnosis had not yet been established. The group without a prior diagnosis experienced a significantly higher complication rate than the group with known CD at the time of surgery [75].

Although the evidence is limited, AGA recommends that rubber band ligation may be considered safe in patients without active rectal inflammation [6,75]. Keighley et al. reported a series of two cases in which rubber band ligation was performed, and one of the patients experienced non-healing lesions, but did not provide information on underlying rectal inflammation [2].

A summary of the clinical manifestations of non-fistulizing PCD and the findings on physical examination is shown in Table 3.

### 6.8. Management Strategy

**Figure 3:**
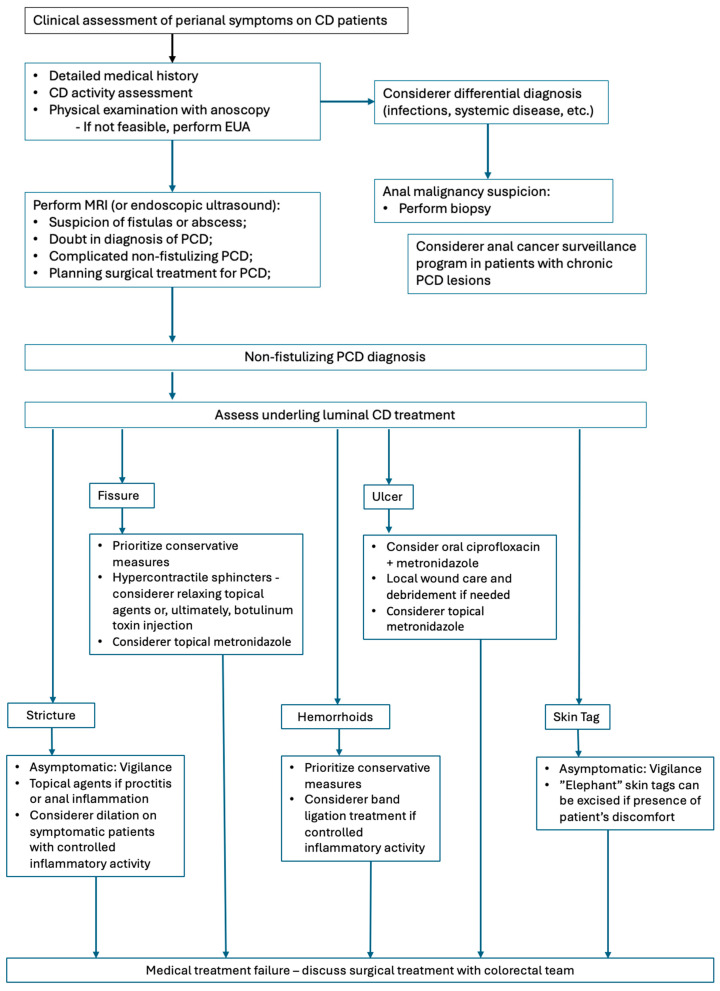
Management of non-fistulizing PCD.

Management of PCD varies considerably in clinical practice, reflecting the heterogeneity of clinical conditions within CD scope, the heterogeneity of patient characteristics, and the availability of resources. Drawing on the literature and expert perspectives, Figure 3 presents an overview of current approaches. It is worth noting that this manuscript consists of a narrative review and the proposed approach represents common considerations in clinical practice, highlighting areas of greater consensus rather than constituting a formal treatment algorithm.

## 7. Conclusions and Future Directions

Non-fistulizing PCD is an underrecognized but clinically significant group of heterogeneous conditions that can be disabling and significantly impact quality of life. Current recommendations on their management are supported by limited evidence based on small retrospective studies or heterogeneous cohort studies, many of which were conducted in the early development of CD comprehension and treatments. Some lesions resolve spontaneously, while others undergo a more challenging healing process, with a risk of progressing to a more complex disease. Beyond individualized clinical evaluation, there is a lack of tools to predict which patients will respond to conservative measures and those at risk of developing complications. In future research, it is important to identify prognostic and risk factors, as well as establish validated instruments that could help predict an unfavorable disease course and support informed decision-making.

Since the introduction of biologic therapy for luminal disease, the natural history of PCD has been altered. When conservative measures fail, surgery may be required, although outcomes are not often optimistic, reinforcing the role of biologics. Further studies are needed to elucidate the optimal timing and combination of biologic and immunomodulatory therapies, as well as to define the role of novel therapeutic agents in disease management. The pursuit of novel biomarkers that could help predict therapeutic response and enable individualized therapies is also a focus area. For refractory, persistent, or complicated cases, a multidisciplinary collaboration among gastroenterologists and colorectal surgeons is essential. As noted, these patients may experience a significant psychological impact, and referral to mental health professionals should be considered an essential part of their management.

Additionally, concerns about a possible association with anal cancer warrant further investigation, including studies assessing the value of anal and perianal cancer screening strategies in these patients. Prospective studies with standardized lesion classification, focusing on disease course monitoring and evaluation of emerging treatments, are essential for establishing updated, evidence-based strategies to improve the management of these patients.

## Figures and Tables

**Table 1:** Incidence of non-fistulizing perianal CD.

Author	Study Design	Country	CD Phenotypes	Population (n)	Time of Follow-Up	Skin Tags	Fissures	Ulcers	Strictures	Hemorrhoids
(Peyrin-Biroulet et al., 2012) [14]	Single-center; Retrospective	United States of America	All phenotypes	310	30 year calculated cumulative risk	32.2%	16%	8.4%	17.5%	NR
(Eglinton et al., 2012) [15]	Single-center; Retrospective	New Zealand	All phenotypes	715	Median 9 years [IQR 2 months–45 years]	11.1%	32.6% ^1^	32.6% ^1^	7.4%	1.6%
(Martínez Sánchez et al., 2022) [16]	Single-center; Retrospective	Spain	All phenotypes	430	12 years	4.6%	30.1%	NR	15%	NR
(Yamamoto et al., 2023) [17]	Multi-center; Prospective	Japan	All phenotypes	673	At the time of diagnosis	19.1%	18.5%	11.1%	4.6%	NR
(Wallenhorst et al., 2016) [12]	Single-center; Prospective	France	All phenotypes	282	5 years	NR	NR	54.6% -Superficial ulcer U1 n = 17; -Cavitating ulcer U2 n = 17;	17.4% -Short stricture S1 n = 32; -Long stricture S2 n = 17;	NR

NR—Not reported; ^1^—Fissures and ulcers were reported together.

**Table 2:** The Cardiff classification, Hughes et al. [24].

The Cardiff Classification		
**U. Ulceration**	**F. Fistula/Abscess**	**S. Stricture**
0. Not present 1. Superficial fissures (a) Posterior and/or anterior (b) Lateral (c) With gross skin tags 2. Cavitating ulcers (a) Anal canal (b) Lower rectum (c) With extension to perineal skin (aggressive ulceration)	0. Not present 1. Low/superficial (a) Perianal (b) Anovulval/anoscrotal (c) Intersphincteric (d) Anovaginal 2. High (a) Blind supralevator (b) High direct (anorectal) (c) High complex (d) Rectovaginal (e) Ileoperineal	0. Not present 1. Reversible stricture (a) Anal canal—spasm (b) Low rectum—membranous (c) Spasm with severe pain—no sepsis identified 2. Irreversible stricture (a) Anal stenosis (b) Extrarectal stricture
Subsidiary Classification (A.P.D.)		
**A. Associated Anal Conditions**	**P. Proximal Intestinal Disease**	**D. Disease Activity (in Anal Lesions)**
0. None 1. Hemorrhoids 2. Malignancy 3. Other(specify)	0. No proximal disease 1. Contiguous rectal disease 2. Colon (rectum spared) 3. Small intestine 4. Investigation incomplete	1. Active 2. Inactive 3. Inconclusive
Simplified Clinical Classification of PCD		
**U. Ulceration**	**F. Fistula/Abscess**	**S. Stricture**
0. Not present	0. Not present	0. Not present
1. Superficial fissure	1. Low/superficial	1. Spasm/membranous
2. Cavitating ulcer	2. High/complex	2. Severe fibrotic

**Table 3:** Clinical manifestations of non-fistulizing PCD.

Lesion	Clinical Presentation	Physical Examination Findings
Fissure	Sharp or burning pain during defecation, possibly lingering afterwards; Rectal bleeding; Pruritus; May be asymptomatic.	Linear/oval ulceration usually limited to dentate line. Multiple and atypically located raises suspicion of association with CD; Can have tenderness to palpation; Chronic cases may show sentinel tag or hypertrophied papilla.
Ulcer	Severe, persistent pain; Anal discharge; Rectal bleeding.	Irregular, deep mucosal defect with indurated edges, granulation tissue, or purulent base; Often off-midline and may involve anoderm, anal canal, or perianal skin.
Skin Tag	Usually asymptomatic; May cause discomfort, pruritus, or hygiene issues.	Two types described: - Large, edematous cyanotic tags (from healed lesions); - Flat, soft “elephant ear” tags.
Stricture	Pain or difficulty during defecation; Feeling of incomplete evacuation; Narrow or ribbon-like stools; Rectal bleeding; Anal discharge; May be asymptomatic.	Narrowed anal canal or tight fibrotic ring; Possible scarring; Resistance or pain on digital examination or anoscope/endoscope insertion.
Hemorrhoids	Painless rectal bleeding; Pruritus; Mucous discharge; Prolapse; Acute pain if thrombosed.	External hemorrhoids: bluish and swelling on anal canal. Internal hemorrhoids: pink-red cushions on anoscopy or visible if prolapsed, reducible or not.
Anal Cancer	Persistent pain, bleeding, discharge; Systemic symptoms if advanced.	Ulcerated, indurated lesion or palpable mass; Non-healing fissure/ulcer; Chronic or long stricture; Possible fixation and regional lymphadenopathy. Superficial inguinal lymphadenopathy can be palpable on physical examination.

## Abbreviations

The following abbreviations are used in this manuscript:

AGA	American Gastroenterological Association
CD	Crohn’s disease
HIV	Human immunodeficiency virus
MRI	Magnetic resonance
PCD	Perianal Crohn’s disease
PDAI	Perianal Disease Activity Index
EUA	Examination under anesthesia
